# Antibiotic Prescribing Habits in Endodontics among Dentists in the Federation of Bosnia and Herzegovina—A Questionnaire-Based Study

**DOI:** 10.3390/antibiotics13090876

**Published:** 2024-09-12

**Authors:** Matea Galić, Ivana Miletić, Tina Poklepović Peričić, Valentina Rajić, Nikolina Nika Većek Jurčević, Ajka Pribisalić, Ivana Medvedec Mikić

**Affiliations:** 1 Private dental practice Grizelj, 88340 Grude, Bosnia and Herzegovina; matea.galic1511@gmail.com; 2 Department of Endodontics and Restorative Dentistry, School of Dental Medicine, University of Zagreb, 10000 Zagreb, Croatia; miletic@sfzg.hr (I.M.); vbrzovic.rajic@sfzg.hr (V.R.); 3 Department of Prosthodontics, Study of Dental Medicine, School of Medicine, University of Split, 21000 Split, Croatia; tina.poklepovic.pericic@mefst.hr; 4 Department of Dental Medicine, University Hospital Center of Split, 21000 Split, Croatia; 5 Department of Public Health, School of Medicine, University of Split, 21000 Split, Croatia; ajka.pribisalic@mefst.hr; 6 The University Department of Health Studies, University of Split, 21000 Split, Croatia; 7 Department of Endodontics and Restorative Dentistry, Study of Dental Medicine, School of Medicine, University of Split, 21000 Split, Croatia

**Keywords:** endodontics, antibiotic prophylaxis, antibiotics, endodontic inflammation

## Abstract

Backgrounds: Antibiotics are used in endodontic treatment to control acute odontogenic infection and for prophylactic purposes. This study aimed to investigate the knowledge of dentists from the Federation of Bosnia and Herzegovina about the choice and the routes of antibiotic administration in endodontics. Methods: This cross-sectional study involved dentists in Federation of Bosnia and Herzegovina health institutions. The Dental Chamber sent a twelve-question survey to members’ email addresses. They were asked about the type, dosage, indications, and side effects of antibiotics used in endodontics. The obtained data were screened and analyzed. Results: A total of 180 questionnaires were filled out. The most commonly prescribed antibiotic was amoxicillin with clavulanic acid. Pulp necrosis with symptomatic apical periodontitis, swelling, and moderately severe symptoms were the main indications for the therapeutic use of antibiotics. Amoxicillin, administered orally at 2 g 1 h before endodontic surgery for patients with bacterial endocarditis, was mostly indicated for the prophylactic use of antibiotics. Conclusions: Based on the results of this study, we can conclude that dentists from the Federation of Bosnia and Herzegovina have limited knowledge regarding antibiotic use in endodontics. Educational activities and campaigns are necessary to raise awareness about antibiotics in dental medicine in the Federation of Bosnia and Herzegovina.

## 1. Introduction

The appropriate clinical use of antibiotics is paramount, as it saves millions of lives every year from various complications of infectious diseases [1]. By eliminating the microorganisms that try to overpower the host’s defense mechanisms and cause disease, antibiotics allow the host to rebuild control, eliminate the microorganisms, and overcome the existing infection [2]. Treating endodontic infections in healthy patients is usually achieved by removing the cause, including chemomechanical root canal treatment and drainage. Sometimes, however, root canal treatment alone is insufficient to control the infection. Still, prescribing antibiotics without the previous steps does not significantly benefit the patient [3].

Dentists account for approximately 10% of antibiotic prescriptions and play a significant role in primary health care [4]. Therefore, their potential influence on reducing bacterial antibiotic sensitivity should not be underestimated [5]. In Croatia, for example, dentists account for 7.41% of the total antibiotic consumption, with penicillin (80%) and cephalosporins (9.4%) being the most commonly prescribed antibiotics [6].

In endodontics, antibiotics are used to control acute odontogenic infection and for prophylactic purposes [2,7]. It is of utmost importance to note that numerous studies like Contaldo et al. [8,9,10] have shown a significant variation among dental medicine doctors in their opinions on when antibiotics are necessary. This variation, coupled with prescribing antibiotics for conditions without indication, such as symptomatic irreversible pulpitis or pulp necrosis, has significantly increased microbial resistance to antibiotics. Therefore, the need for appropriate antibiotic-prescribing practices among dentists is urgent and cannot be overstated. It is a matter of public health and patient safety.

In 2017, the European Society of Endodontology (ESE) published clinical practice guidelines [11] covering indications for the therapeutic use of antibiotics in endodontics. The guidelines were developed based on evidence from numerous studies and are crucial to help clinicians, including dentists, to make informed decisions about when, how, and how much antibiotics to prescribe, guiding their practice and ensuring patient safety.

Antibiotics are recommended as an additional therapeutic agent in cases of acute apical abscesses with pronounced general symptoms, including elevated body temperature, malaise, trismus, lymphadenopathy, and fever [12,13]. In addition, the indications for the use of antibiotics are a rapid course of the disease and sudden worsening of symptoms (within 24 to 48 h), diffuse swellings, and acute apical abscesses in immunocompromised patients with reduced defense mechanisms (diabetics, patients on corticosteroid and immunosuppressive therapy, and patients with severe immunological diseases).

Antibiotic prophylaxis in endodontics is recommended for interventions considered at risk for developing bacteriemia. Prophylaxis aims to prevent postoperative local infection and the systemic spread of bacteria from the oral cavity to different body parts in high-risk patients [14,15]. Interventions include manipulations of the gingiva, instrumentation over the apex, periapical surgery, and perforations of the oral mucosa, including scaling and root canal procedures, but not non-invasive dental procedures, such as restorative procedures, infiltration, and conductive anesthesia [15].

Antibiotic prophylaxis is recommended for immunocompromised patients regardless of the cause of their immunodeficiency, including those with leukemia, AIDS, chronic diseases like end-stage renal disease, patients on dialysis, those with uncontrolled diabetes, those receiving chemotherapy, radiation, steroids, or immunosuppressive post-transplant medications, and those with genetic defects [8]. The new, updated European Society of Cardiology (ESC) guidelines recommend antibiotic prophylaxis to prevent infective endocarditis in patients with damaged or artificial valvules or with a previous history of infective endocarditis [16]. Patients receiving head and neck radiotherapy and those on intravenous bisphosphonates also require antibiotic prophylaxis [15]. If in doubt, the patient’s doctor should be consulted before endodontic treatment [9]. One hour before treatment, two grams of amoxicillin should be used for prophylactic purposes.

Amoxicillin is the most commonly used penicillin-type antibiotic in endodontics. With the adjunct of clavulanic acid, amoxicillin is the antibiotic of choice for the causative agents of odontogenic infections that produce B-lactamase and has been proven to be effective in the treatment of endodontic infections, especially in immunocompromised patients [13]. Clindamycin is the treatment of choice for patients allergic to penicillin, along with clarithromycin and azithromycin. In cases of deterioration of the clinical symptoms despite the use of antibiotics after 24 to 48 h, the dose of the applied antibiotic is not increased; instead, an additional antibiotic, such as metronidazole, is prescribed [13]. Metronidazole has been proven to be effective in treating anaerobic infections, and in combination with penicillin antibiotics, it works synergistically.

However, antibiotics do have some side effects, the most common of which are nausea and diarrhea, skin rash, vaginal candidiasis, and sometimes a combination of these [17,18].

This questionnaire-based study aimed to explore the antibiotic prescribing habits among dentists in the Federation of Bosnia and Herzegovina for therapeutic and prophylactic purposes in endodontics. We hypothesize that the knowledge of dentists from the Federation of Bosnia and Herzegovina about the choice of antibiotics and the diagnoses that require antibiotics for treatment and prophylactic purposes in endodontics is suboptimal, regardless of the degree level and type of practice.

## 2. Results

Out of 180 dentists who completed the online questionnaire, most were female (58.9%), with a median age of 37 and a median length of experience of 8 years (Table 1).

Regarding the therapeutic use of antibiotics in patients without a penicillin allergy, 50.6% of questioned doctors reported prescribing amoxicillin in combination with clavulanic acid (875 mg/125 mg). The differences in antibiotic prescription were statistically significant (X^2^ = 244.5, *p* < 0.001) (Table 2). 

For patients with reported penicillin allergies, the majority of dentists prescribed clindamycin (33.9%), followed by lincomycin (29.4%), erythromycin (17.2%), metronidazole (10.0%), and azithromycin (9.4%).

The distribution of answers to the question concerning specific clinical conditions when one would prescribe antibiotics, both for treatment and prophylaxis, is presented in Table 3.

Answers to the question concerning medical conditions that require antibiotic prophylaxis are presented in Table 4.

The answers varied when asked about the doses and administration of antibiotics for prophylactic purposes. Most dentists (57.2%) reported prescribing 2 g of amoxicillin orally one hour before the procedure. Other dentists, however, reported using different doses and at different times (Table 5). This difference was statistically significant (X^2^ = 48.6, *p* < 0.001).

The most frequently reported side effects of antibiotics were nausea and diarrhea (48.9%) and skin rash (42,8%). Other side effects were reported in fewer responses: oral candidiasis (24,4%), vaginal candidiasis (17.8%), and metallic taste (11.1%). Since subjects could report more than one side effect, the exact distribution of responses is listed in Table 6. The differences in these responses were statistically significant (X^2^ = 144.8, *p* < 0.001) (Table 6).

## 3. Discussion

From the results of this study, one can see that most dentists from the Federation of BiH, regardless of their work experience, prescribe amoxicillin in combination with clavulanic acid as the first choice for treating endodontic infections. Groups of authors from Croatia [19,20], Brazil [21], Israel, and the former Soviet Union [22] published similar data. The common prescription of this type of antibiotic can be linked to two things. One is that amoxicillin, combined with clavulanic acid, controls odontogenic infections or their causative agents well. The second is the knowledge and habits that doctors bring from their studies. Very often, the knowledge learned is not questioned but applied in practice. However, some dentists still choose clindamycin as a first choice for treating odontogenic infection, even if the patients are not allergic to penicillin. It is necessary to emphasize that none of the above adheres to the guidelines provided by professional expert societies [9,15].

The correct use of antibiotics in dental medicine is crucial to balancing the need for antibiotics to help treat odontogenic infections and reduce bacterial resistance to their action. This goal is achieved using antibiotics only when indicated at the correct dose [8].

As for patients with a penicillin allergy, clindamycin was the first treatment of choice for endodontic infections for more than one-third of our respondents, followed by lincomycin. Erythromycin was only the third choice for our respondents; it is considered the most common substitute for clindamycin, according to a worldwide study by Segura-Egea and colleagues in 2017 [5].

Almost all participants in our study know the main indication for the adjuvant therapeutic use of antibiotics. Still, a certain number of dentists consider symptomatic irreversible pulpitis with acute apical periodontitis and moderate/severe symptoms as a justified reason for systemic antibiotics. In studies carried out by Segura-Egea et al. [5] and Sovic et al. [23], similar results were found. In an American national survey from 2017, the authors highlighted that antibiotics continue to be prescribed in clinical situations when they are typically not indicated, most commonly because of patient expectations and pain [24].

This unjustified prescription in our and other studies may be due to the lack of knowledge and confidence in treating endodontic infections, which can be a critical reason for the overuse of antibiotics in dental medicine [25].

Considering this study’s nature, which relies on the participants’ self-reports and is affected by subjectivity in answers, the results may underestimate the actual antibiotic prescription practices. Objective assessments of real-world data from electronic health systems might even show higher rates of antibiotic use, especially for asymptomatic conditions like necrotic pulp or pain without symptoms.

More than half of the dentists in our study believe that the only medical condition requiring antibiotic prophylaxis is recovered infective endocarditis. Other conditions that have been well specified in the available guidelines of the European Endodontic Society [9,15] are known to only one-third of dentists. Our results, however, show that there are still a significant number of dentists, more than one-third, who believe that no dental procedure requires antibiotic prophylaxis, which is quite dangerous for patients whose medical conditions require protection from potential bacteriemia resulting from dental procedures. These data and the lack of knowledge about dental procedures requiring antibiotic prophylaxis are alarming.

Participants’ answers about antibiotics’ most common side effects show they are quite familiar with them. Gastrointestinal side effects are, in most cases, connected with clavulanic acid [23,26]. For metronidazole, only rare cases were found across a broad spectrum of reactions, including allergic contact dermatitis [27]. Although it is generally thought that vulvovaginal candidiasis (VVC) is common after systemic antibiotic therapy, with gynecology or medical textbooks containing the statement that Candida vaginitis may result from antibiotic treatment, only a few investigators have studied the incidence of VVC related to antibiotic use (antibiotic-associated VVC), and even fewer have examined the associated contributing risk factors [28].

Since no research has been conducted so far in the Federation of Bosnia and Herzegovina, this study is essential as it provides insights into the knowledge and habits of dental medicine doctors in this region regarding antibiotic use for endodontic purposes.

Our study results show an urgent need for specifically tailored educational strategies targeted at dental medicine doctors based on the best available evidence and aligned with the available high-quality recommendations. Knowledge translation can bridge the generational knowledge gap, allowing better uptake of the research evidence and the use of current clinical practice guideline recommendations.

There are some limitations to this study, however. Although the cross-sectional study design that we applied is best suited to answering the questions concerning current practices on antibiotic use, we acknowledge that the nature of the data collection allows the introduction of certain confounding factors and the potential to misinterpret the study results. We were therefore cautious when interpreting the study findings, since the use of an electronic questionnaire may have influenced the results. First, the sample’s representativeness relies on dentists’ willingness to answer an online survey. We acknowledge that we might have included only those with higher computer skills and more frequent use of computers in general, likely a younger population. This explains why most dentists in our study were women in their late thirties with less than ten years of work experience. These individuals may use email communication more frequently and are thus more eager to respond to online surveys. Bolfoni et al. found similar results in their study, which surveyed Brazilian endodontists [21]. This is interesting, however, because it reveals a lack of knowledge among new dental medicine doctors. The limitation concerning online data collection may imply that our results could be somewhat different from the practice in the target population. Still, the sample size and the fact that the Chamber distributed the questionnaire among all their members might have helped the sample’s representativeness. Data on dentists’ level of degree, type of practice, and the number of days that antibiotics were prescribed were not collected, which also represents a limitation of this study, since the given prescription guidelines may or may not have varied over the years.

The number of dentists completing the survey is under the precalculated sample size but is large enough to show a trend in the population. Future research should encompass representative study samples in size and sample diversity and apply objective measurement instruments, like the analysis of prescribed antibiotics with indications from the health system records. In practical terms, the results of this study call for educational activities to raise awareness about the appropriate use of antibiotics and for the more rational use of antibiotics in line with the available recommendations.

## 4. Materials and Methods

### 4.1. Study Design

This cross-sectional, questionnaire-based study involved a convenient sample of dental medicine doctors working in Federation of Bosnia and Herzegovina health institutions. Before starting the survey, we used the Epitools sample size calculator (available at https://epitools.ausvet.com.au/samplesize, accessed on 2 February 2023) for power analysis. With the estimated population size of 700, a confidence level of 95%, a margin of error of 5%, and a response distribution of about 30%, we estimated that the minimum sample size for this study would be 222.

We created an online questionnaire in Croatian, one of three official languages in the Federation of Bosnia and Herzegovina, using Google Forms and shared it with the Dental Chamber of the Federation of Bosnia and Herzegovina. The Dental Chamber of the Federation of Bosnia and Herzegovina is the authoritative body for dentists in the Federation of Bosnia and Herzegovina. Its members are dentists who are licensed to work by the Chamber. Professional societies are responsible for creating work guidelines, and the Chamber ensures that courses and workshops are held where these guidelines are presented and where leading experts from a specific field educate colleagues.

All registered members of the Dental Chamber of the Federation of Bosnia and Herzegovina were eligible for inclusion in this study. The exclusion criterion was questionnaires not being fully filled out. Upon approval from the board, the Chamber then distributed the questionnaire to the email addresses of all its members. This study was conducted from March to April 2023 and was reported following the Strengthening the Reporting of Observational Studies in Epidemiology (STROBE) reporting checklist [29]. The Faculty of Medicine of the University of Mostar Ethics Committee approved this study.

### 4.2. Questionnaire

The introductory part of the questionnaire contained information about the scope of the study for dentists to read before answering the questions. The questionnaire consisted of 12 questions. The first set referred to gender, age, and years of experience as a dentist. The second section of the questionnaire assessed dentists’ knowledge of antibiotic selection and dosage for endodontic treatments and prophylaxis. Participants were presented with various clinical scenarios and were asked to choose from predefined response options, resulting in the collection of nominal data. Completing the questionnaire was considered consent for participation. The author compiled the questionnaire. Validation was performed according to the paper of Boparai et al. [30]. The expert team (two endodontics specialists (I.M.M., I.M) and two general dentists (N.N.V.J., M.G)) commented on and corrected the questionnaire, and the final version was compiled. The questionnaires were tested on a test sample of ten dentists. The time required for completion was measured, and comments on the comprehensibility of the questions were recorded. The expert team reviewed the comments and made the necessary corrections, completing the validation of the questionnaire. The internal consistency of test items was measured using Cronbach α = 0.78. Pre–post test results are not applicable since we did not implement any intervention; this study aimed to analyze data on current practices.

### 4.3. Data Analysis

All answers from the test were automatically imported into an Excel file (Ver. Office 2007, Microsoft, Redmond, Washington, DC, USA). We coded the answers and analyzed the data using the SPSS V.24 (IBM, Armonk, New York, NY, USA). The normality of the data distribution was checked with the Kolmogorov–Smirnoff test. We used descriptive statistics to present data, frequencies with percentages (%) for the categorical data, and median with interquartile range (IQR) for numerical data. The chi-square test (X^2^) was used to investigate the differences between the categorical variables with a significance level of *p* < 0.05.

## 5. Conclusions

The knowledge of dentists from the Federation of Bosnia and Herzegovina about the choice of antibiotics and the diagnoses that require antibiotics for treatment and prophylactic purposes in endodontics is suboptimal. Educational activities and campaigns aimed at raising awareness about the use of antibiotics in dental medicine are necessary.

## Figures and Tables

**Table 1:** Demographic characteristics of the respondents.

Female; n (%)	106 (58.9)
Age (years); median (interquartile range (IQR))	37.0 (15.0)
Length of experience (years); median (IQR)	8.0 (13.0)

**Table 2:** Antibiotic prescription for therapeutic use in endodontics.

Antibiotic	n	%	*p*-Value
Amoxicillin	60	33.3	<0.001 *
Amoxicillin + clavulanic acid	91	50.6	
Clindamycin	11	6.1	
Azithromycin	7	3.9	
Metronidazole	11	6.1	

n—number; %—percentage; *—X^2^ test.

**Table 3:** Indications for therapeutic and prophylactic use of antibiotics in endodontics.

Indications for AT	n	%	*p*-Value
Pulp necrosis with symptomatic apical periodontitis, swelling and moderately severe symptoms	164	91.1	
Pulp necrosis without symptoms and present sinus tract	5	2.8	<0.001 *
Symptomatic irreversible pulpitis with acute apical periodontitis	11	6.1	
**Indications for AP**			
Before endodontic surgery	97	53.9	
No dental procedures require antibiotic prophylaxis	70	38.9	<0.001 *
Before non-surgical procedures	13	7.2	

AT—antibiotics for therapeutic use; AP—antibiotics for prophylactic use; n—number; %—percentage; *—X^2^ test.

**Table 4:** Medical conditions that require antibiotic prophylaxis before endodontic procedures.

Patient’s Medical Condition	n	%	*p*-Value
Infective endocarditis only	99	55	
Immunosuppressed or medically compromised patients, patients at risk of infective endocarditis, patients with artificial joints, patients with head and neck tumors under radiotherapy	59	32.8	<0.001 *
Immunosuppressed or medically compromised patients, patients at risk of infective endocarditis	22	12.2	

n—number; %—percentage; *—X^2^ test.

**Table 5:** Antibiotic schemes that dentists use for prophylactic use of antibiotics in endodontics.

Antibiotic Scheme	n	%	*p*-Value
Amoxicillin, administered orally in a dose of 2 g 1 h before treatment	103	57.2	<0.001 *
Amoxicillin is administered orally at 1 g 1 h before treatment.	30	16.7	
Amoxicillin, administered orally in a dose of 1 g 1 h before and 1 g after treatment	47	26.1	

n—number; %—percentage; *—X^2^ test.

**Table 6:** Side effects of antibiotics according to dentists in this study.

Side Effects of Antibiotics	n	%	*p*-Value
Oral candidiasis	16	8.9	<0.001 *
Vaginal candidiasis	11	6.1	
Nausea and diarrhea	36	20.0	
Skin rash	35	19.4	
Metallic taste	7	3.9	
Oral candidiasis, vaginal candidiasis	3	1.7	
Oral candidiasis, nausea and diarrhea	19	10.6	
Vaginal candidiasis, nausea and diarrhea	11	6.1	
Nausea and diarrhea, Skin rash	17	9.4	
Nausea and diarrhea, skin rash, metallic taste	2	1.1	
Oral candidiasis, vaginal candidiasis, Nausea and diarrhea, skin rash	1	0.6	
Skin rash, metallic taste	11	6.1	
Vaginal candidiasis, skin rash, nausea and diarrhea	2	1.1	
Vaginal candidiasis, skin rash	4	2.2	
Oral candidiasis, skin rash	5	2.8	

n—number; %—percentage; *—X^2^ test.

## Data Availability

Data are available from the corresponding author upon request.

## References

[1] Moser C., Lerche C.J., Thomsen K., Hartvig T., Schierbeck J., Jensen P.Ø., Ciofu O., Høiby N. (2019). Antibiotic therapy as personalized medicine—General considerations and complicating factors. APMIS.

[2] Mues N., Chu H.W. (2020). Out-Smarting the Host: Bacteria Maneuvering the Immune Response to Favor Their Survival. Front. Immunol..

[3] Longman L.P., Preston A.J., Martin M.V., Wilson N.H.F. (2000). Endodontics in the adult patient: The role of antibiotics. J. Dent..

[4] Domínguez-Domínguez L., López-Marrufo-Medina A., Cabanillas-Balsera D., Jiménez-Sánchez M.C., Areal-Quecuty V., López-López J., Segura-Egea J.J., Martin-González J. (2021). Antibiotics Prescription by Spanish General Practitioners in Primary Dental Care. Antibiotics.

[5] Segura-Egea J.J., Martin-Gonzalez J., Jimenez-Sanchez M.D.C., Crespo-Gallardo I., SaucoMarquez J.J., Velasco-Ortega E. (2017). Worldwide antibiotic prescription pattern in endodontic infections. Int. Dent. J..

[6] Bebek B. (2012). The Contribution of Doctors of Dental Medicine in the National Outpatient Consumption and Prescription of Antibiotics in the Republic of Croatia.

[7] Bansal R., Jain A., Goyal M., Singh T., Sood H., Malviya H.S. (2019). Antibiotic abuse during endodontic treatment: A contributing factor to antibiotic resistance. J. Family Med. Prim. Care.

[8] Contaldo M., D’Ambrosio F., Ferraro G.A., di Stasio D., di Palo M.P., Serpico R., Simeone M. (2023). Antibiotics in Dentistry: A Narrative Review of the Evidence beyond the Myth. Int. J. Environ. Res. Public Health.

[9] Cope A.L., Wood F., Francis N.A., Chestnutt I.G. (2015). General practitioners’ attitudes towards the management of dental conditions and use of antibiotics in these consultations: A qualitative study. BMJ Open.

[10] Palmer N. (2000). A comparison of antibiotic prescription by doctors and dentists for acute dental conditions. Br. Dent. J..

[11] Segura-Egea J.J., Gould K., Sen B.H., Jonasson P., Cotti E., Mazzoni A., Sunay H., Tjäderhane L., Dummer P.M.H. (2017). Antibiotics in Endodontics—A review. Int. Endod. J..

[12] Stein K., Farmer J., Singhal S., Marra F., Sutherland S., Quiñonez C. (2018). The use and misuse of antibiotics in dentistry: A scoping review. J. Am. Dent. Assoc..

[13] Santini M., da Rosa R.A., Ferreira M.B., Barletta F., Longo do Nascimento A., Weissheimer T., Estrela C., So M.V. (2021). Medications Used for Prevention and Treatment of Postoperative Endodontic Pain: A Systematic Review. Eur. Endod. J..

[14] Chambers J.B., Thornhill M., Shanson D., Prendergast B. (2016). Antibiotic prophylaxis of endocarditis: A NICE mess. Lancet Infect. Dis..

[15] Segura-Egea J.J., Gould K., Şen B.H., Jonasson P., Cotti E., Mazzoni A., Sunay H., Tjäderhane L., Dummer P.M. (2018). European Society of Endodontology position statement: The use of antibiotics in endodontics. Int. Endod. J..

[16] Delgado V., Ajmone Marsan N., de Waha S., Bonaros N., Brida M., Burri H., Caselli S., Doenst T., Ederhy S., Erba P.A. (2023). ESC Scientific Document Group. 2023 ESC Guidelines for the management of endocarditis. Eur. Heart J..

[17] Gillies M., Ranakusuma A., Hoffmann T., Thorning S., McGuire T., Glasziou P., del Mar C. (2015). Common harms from Amoxicillin: A systematic review and meta-analysis of randomized placebo-controlled trials for any indication. CMAJ.

[18] Faye A.S., Allin K.H., Iversen A.T., Agrawal M., Faith J., Colombel J.F., Jess T. (2023). Antibiotic use as a risk factor for inflammatory bowel disease across the ages: A population-based cohort study. Gut.

[19] Šutej I., Lepur D., Božić D., Pernarić K. (2021). Medication Prescribing Practices in Croatian Dental Offices and Their Contribution to National Consumption. Int. Dent. J..

[20] Petrac L., Gvozdanovic K., Perkovic V., Petek Zugaj N., Ljubicic N. (2024). Antibiotics Prescribing Pattern and Quality of Prescribing in Croatian Dental Practices-5-Year National Study. Antibiotics.

[21] Bolfoni M.R., Pappen F.G., Pereira-Cenci T., Jacinto R.C. (2018). Antibiotic prescription for endodontic infections: A survey of Brazilian Endodontists. Int. Endod. J..

[22] Solomonov M., Batashvili G., Shuster A., Slutzky H., Moshonov J., Buchkovskii O., Lvovsky A., Azizi H., Levin A., Ben Itzhak J. (2023). Systemic antibiotics in endodontics-International questionnaire study: Choice of types, dosage, loading and duration. Aust. Endod. J..

[23] Sović J., Šegović S., Tomasić I., Pavelić B., Šutej I., Anić I. (2020). Antibiotic Administration Along with Endodontic Therapy in the Republic of Croatia: A Pilot Study. Acta Stomatol. Croat..

[24] Germack M., Sedgley C.M., Sabbah W., Whitten B. (2017). Antibiotic Use in 2016 by Members of the American Association of Endodontists: Report of a National Survey. J. Endod..

[25] Aminoshariae A., Kulild J.C. (2016). Evidence-based recommendations for antibiotic usage to treat endodontic infections and pain: A systematic review of randomized controlled trials. J. Am. Dent. Assoc..

[26] Huttner A., Bielicki J., Clements M.N., Frimodt-Møller N., Muller A.E., Paccaud J.P., Mouton J.W. (2020). Oral amoxicillin and amoxicillin-clavulanic acid: Properties, indications and usage. Clin. Microbiol. Infect..

[27] Dilley M., Geng B. (2022). Immediate and Delayed Hypersensitivity Reactions to Antibiotics: Aminoglycosides, Clindamycin, Linezolid, and Metronidazole. Clin. Rev. Allergy Immunol..

[28] Lopes J.P., Lionakis M.S. (2022). Pathogenesis and virulence of *Candida albicans*. Virulence.

[29] Von Elm E., Altman D.G., Egger M., Pocock S.J., Gøtzsche P.C., Vandenbroucke J.P. (2008). STROBE Initiative The strengthening the reporting of observational studies in epidemiology (STROBE) statement: Guidelines for reporting observational studies. J. Clin. Epidemiol..

[30] Boparai J.K., Singh S., Kathuria P. (2018). How to Design and Validate A Questionnaire: A Guide. Curr. Clin. Pharmacol..

